# From Plant to Polymers: Micro-Processing Sisal Fiber-Reinforced PLA/PHA Bio-LFTs at Laboratory Scale

**DOI:** 10.3390/polym17121618

**Published:** 2025-06-11

**Authors:** Rumeysa Yıldırım, Nursel Karakaya, Bas Liebau, Tim Welten, Beyza Bayram, Mehmet Kodal, Güralp Özkoç

**Affiliations:** 1 Department of Chemical Engineering, Kocaeli University, Kocaeli 41001, Türkiye; rumeysa.yildirim@kocaeli.edu.tr (R.Y.); beyzabayram662@gmail.com (B.B.); 2 Xplore Instruments B.V., 6135 KT Sittard, The Netherlands; nursel.karakaya@xplore-together.com (N.K.); bas.liebau@xplore-together.com (B.L.); tim.welten@xplore-together.com (T.W.); 3 Sabancı University Nanotechnology Research and Application Center, Istanbul 34956, Türkiye; 4 Polymer Science and Technology Graduate Program, Kocaeli University, Kocaeli 41001, Türkiye; 5 Department of Chemistry, Istinye University, Istanbul 34396, Türkiye

**Keywords:** bio-LFT, PLA, PHA, thermoplastic composites, microcompounding

## Abstract

This study explores the development of long fiber-reinforced thermoplastic (LFT) composites based on blends of poly(lactic acid) (PLA) and polyhydroxyalkanoate (PHA), reinforced with sisal fibers. A novel lab-scale LFT line was employed to fabricate the long fiber composites, effectively addressing the challenges associated with dispersing and processing high-aspect-ratio natural fibers. The rheological, mechanical, thermal, and morphological properties of the resulting bio-LFT composites were systematically characterized using FTIR, SEM, rotational rheology, mechanical testing, DSC, and TGA. The results demonstrated generally homogeneous fiber dispersion, although limited interfacial adhesion between the fibers and polymer matrix was observed. Mechanical tests revealed that sisal fiber incorporation significantly enhanced tensile strength and stiffness, while impact toughness decreased. Thermal analyses showed improved crystallinity and thermal stability with increasing PHA content and fiber reinforcement. Overall, this work highlights the potential of natural fibers to create high-performance, sustainable biocomposites and lays a solid foundation for future advancements in developing eco-friendly structural materials.

## 1. Introduction

Extrusion and injection molding represent two of the most extensively employed processing methods for the fabrication of fiber-reinforced polymer composites. The overall mechanical performance of the composite material is strongly influenced by factors such as fiber dispersion and orientation [1,2,3]. As described by classical mechanics [1,4], the mechanical load imposed on a fiber-reinforced composite is transmitted from the polymer matrix to the reinforcing fibers via shear stresses at the fiber–matrix interface. This stress transfer is most effective when the fibers are aligned in the direction of the applied load, with efficiency further enhanced by an increased fiber aspect ratio, defined as the ratio of fiber length (L) to diameter (D). Therefore, long fiber-reinforced thermoplastic (LFT) composites have gained considerable prominence, especially in the automotive and transportation industries, due to their advantageous combination of mechanical performance, processability, and recyclability. These composites exhibit a high specific modulus and strength, excellent impact and corrosion resistance, and virtually indefinite shelf life. Such properties have driven their increasing use in diverse engineering applications, positioning LFTs among the most advanced lightweight structural materials available today [5,6]. Glass fibers are the most widely utilized reinforcement material in long fiber-reinforced thermoplastic composites, attributed to their low cost, ease of processing, and favorable mechanical properties. Among these, glass fiber-reinforced polypropylene is the most extensively studied system, primarily due to its extensive application within the automotive industry [5,7,8,9].

To mitigate environmental impact and advance the development of sustainable materials, there has been a growing interest in fibers derived from biological sources in recent years [10,11]. Among these, plant-based fibers, such as jute [12], kenaf [13], flax [14], bamboo [15], sisal, and hemp [16], have been widely adopted in the production of composite materials. Although natural fiber-reinforced polymer composites have not traditionally been classified as LFTs, growing interest has emerged in the development of composites incorporating long natural fibers [5]. In a study conducted by Okutan Baba and Özmen [17], the mechanical properties of green composites fabricated by incorporating chicken feather fibers (CFF) into a poly(lactic acid) (PLA) matrix were investigated. Fibers with lengths of 0.3 cm and 2 cm were classified as short and long fibers, respectively. The authors stated that CFF/PLA composites reinforced with long fibers exhibit higher compressive modulus and strength compared to those reinforced with short fibers. In another study, the pultrusion technique, which facilitates the fabrication of composite pellets containing relatively long fibers, was utilized to produce long fiber pellets (LFT) for injection molding applications by Wang et al. [18]. Specifically, continuous jute yarns were continuously drawn through a PLA resin bath and subsequently passed through a heated die, allowing for effective resin impregnation. After sufficient cooling, the impregnated yarns were chopped into pellets. To mitigate this limitation, a re-compounding process was employed to produce re-compounded pellets (RP). Three material types—LFT, RP, and a 50:50 hybrid mixture of LFT and RP (denoted as LFT/RP)—were then used to fabricate dumbbell-shaped test specimens incorporating weld lines. They observed that the fiber separation and dispersion in the re-compounded pellets were superior to those in the original long fiber pellets, leading to enhanced interfacial bonding and improved mechanical properties in the injection-molded jute/PLA composites. In another study, Torres-Giner et al. [19] investigated mechanical and thermomechanical properties of pita fiber-reinforced poly(3-hydroxybutyrate) (PHB) composites prepared by compression molding. The authors reported that the incorporation of 40 wt.% 20-mm-long pita fibers into PHB composite sheets resulted in an approximately 55% increase in elastic modulus compared to unreinforced PHB sheets.

The predominant method for producing LFT pellets is the hot melt impregnation process. In this technique, collimated continuous fiber tows are drawn through an impregnation die, where they are impregnated with molten thermoplastic polymer extruded from a conventional screw extruder [6]. Upon exiting the die, the impregnated tow is rapidly cooled, pulled through a pulling system, and subsequently chopped into pellets of defined lengths, typically between 5 and 50 mm [6,20]. These pellets are subsequently processed into final components using conventional manufacturing techniques, most notably injection molding and extrusion-compression molding. In the injection molding process, LFT pellets are first melted in an extruder and then injected into a closed mold cavity under high pressure, where they solidify into the desired shape. This process induces significant fiber orientation along the flow direction, thereby enhancing the mechanical properties of the molded part in that direction [6,21,22].

The development of biopolymer blends that fulfill the technical requirements of target applications while maintaining a favorable cost-to-performance ratio remains a critical objective in sustainable materials research. Among the spectrum of biodegradable polymers derived from renewable resources, polyhydroxyalkanoates (PHAs) demonstrate mechanical properties that position them as promising alternatives to a broad range of petroleum-based polymers, particularly in high-performance sectors such as the automotive industry. However, the widespread adoption of PHAs is currently constrained by their high production costs, rendering them economically unfeasible for large-scale, cost-sensitive applications. Conversely, poly(lactic acid) (PLA) represents a more cost-effective biopolymer; however, it does not fully satisfy the demanding performance criteria of applications such as automotive components, particularly in terms of thermal resistance [23]. Moreover, both PHA and PLA exhibit limited impact toughness, which restricts their applicability in environments requiring high mechanical resilience [24,25,26]. To achieve a viable alternative to conventional polymers, it is imperative to address these limitations by reducing production costs while enhancing thermal and mechanical performance. One promising strategy involves the formulation of polymer blends, specifically combining PHA with the more economically viable PLA. This approach aims to leverage the complementary properties of each biopolymer to develop materials that offer a more balanced and application-ready solution. Moreover, the addition of natural fibers to PLA/PHA blends can improve the overall mechanical properties. In recent years, there has been a marked increase in interest toward the development of sisal fiber-reinforced composites, driven by the fiber’s low cost, low density, high specific strength and modulus, biodegradability, non-toxicity, and renewability [27].

Despite the mechanical benefits associated with the use of long plant fibers, such as sisal, their application in extrusion processing is limited. High-aspect-ratio plant fiber bundles tend to entangle during processing, resulting in poor dispersion within the polymer matrix [28]. Additionally, their low bulk density and extended length complicate feeding into screw extruders, particularly during the melting or injection stages [4]. Consequently, plant fibers are more commonly incorporated in powder form or as short fibers, typically less than 10 mm in length [1]. Therefore, to eliminate these disadvantages, for the first time in the literature, long sisal fiber-reinforced LFT-PLA/PHA bio-composite materials were prepared via the micro-processing method in this study. The rheological and mechanical properties of the bio-LFT composites were investigated. Moreover, the dispersion of sisal fiber in the PLA/PHA matrix was judged by scanning electron microscopy. This study provides a good starting point for discussion and further research on natural fiber-reinforced bio-composites.

## 2. Materials and Methods

### 2.1. Materials

Poly(lactic acid) (PLI005) with a melt flow index (MFI) of 16 g/10 min at 190 °C/2.16 kg was obtained from NaturePlast, France. The average molecular weight of the PLA was measured as 106,000 Da and heterogeneity as 1.97 using gel permeation chromatography (GPC). Polyhydroxyalkanoate (PHA) was also purchased from NaturePlast and has an MFI of 5–18 g/10 min (at 190 °C/2.16 kg). Continuous braided sisal fiber was obtained from the local market under the trade name of Xpose.

### 2.2. Processing

**Figure 1:**
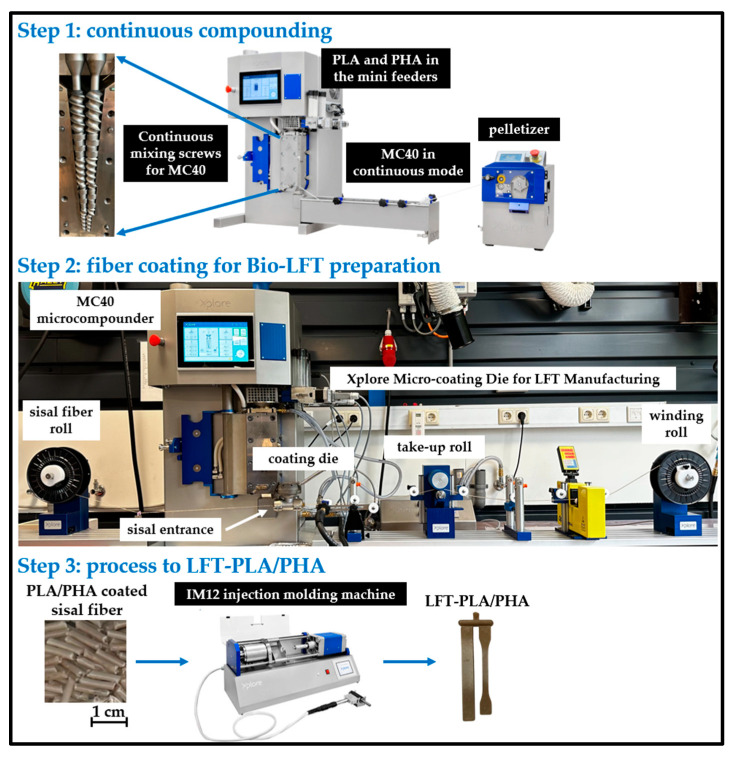
Preparation steps of LFT-PLA/PHA samples.

The LFT compounds were prepared in multiple steps (see Figure 1). In the first step, PLA and PHA were compounded using a 40 mL co-rotating microcompounder (MC40, Xplore Instruments BV, The Netherlands) in continuous mode. The barrel temperature was set to 180 °C, and the screw speed was maintained at 50 rpm. PLA and PHA granules were fed separately via volumetric mini feeders to achieve PLA/PHA weight ratios of 30/70, 50/50, and 70/30. The throughput rate was maintained at 3.5 kg/h. The extruded strand was subsequently cooled in a water bath and pelletized using a pelletizer (Xplore Instruments BV, The Netherlands).

In the second step, sisal fiber was coated with the PLA/PHA compound to produce LFT granules using a micro-coating line (Xplore Instruments BV, The Netherlands). This setup included a tension-controlled de-winding unit through which the sisal fiber passed into the bore of a coating die. The MC40 was again used to melt and feed the compound from the top of the coating die. A take-up unit regulated the line speed at 3 m/min. The coated strand was then wound onto a spool.

In the third step, the PLA/PHA-coated sisal filament was chopped into 1 mm granules to produce bio-LFT. Under these conditions, the fiber content was found to be 15%.

To prepare test samples, the bio-LFT granules were fed back into the MC40, compounded for 1 min, and immediately transferred to a lab-scale injection molding machine (IM12, Xplore Instruments BV, The Netherlands). The melt temperature was maintained at 180 °C, the mold temperature at 25 °C, and the injection molding air pressure and holding time were set to 10 bar and 5 s, respectively.

### 2.3. Characterization

The possible interactions between PLA, PHA, and sisal fiber were investigated via a Perkin Elmer Spectrum 100 model Fourier transform infrared (FTIR) spectroscopy device. The specimens were placed in an attenuated total reflection (ATR) unit on a diamond crystal, and spectra in the 4000–650 cm^−1^ wavenumber range were acquired.

Rheological behavior was assessed using an Anton Paar MCR 102 rheometer with a parallel plate configuration. Frequency sweep tests were conducted at a constant temperature of 185 °C under a nitrogen atmosphere. The shear strain was fixed at 1%, and the angular frequency varied from 0.1 to 600 rad/s.

The sisal fiber length distributions were determined from injection-molded tensile test specimens. The samples were dissolved in 50 mL of chloroform at 50 °C for 20 min. After dissolution, the fibers were separated via filtration and subsequently dried. A Nikon LV100 POL optical microscope was employed to measure the extracted fibers at 5× magnification. The measurements were conducted with Nikon Imaging Software (NIS-Elements, Version 4.20), employing contrast-based differentiation to accurately delineate fiber boundaries. Non-linear fiber geometries were appropriately considered throughout the measurement process. Length distribution curves were obtained based on measurements of up to 400 individual fibers.

The interfacial topology of PLA/PHA and LFT-PLA/PHA samples was observed using a JEOL JIB 450 scanning electron microscope (SEM). SEM images were taken of cryogenically fractured surfaces of the impact specimens. Prior to analysis, the samples were coated with a thin layer of gold to eliminate arching.

Mechanical properties were evaluated through tensile and Izod impact tests. A minimum of five specimens were tested for each group, and the average values were reported along with the corresponding standard errors. Tensile testing was performed in accordance with ISO 527-5A using an Instron Model 3345 universal testing machine at a crosshead speed of 5 mm/min. The Izod impact strength of samples with a 2 mm V-notch was measured using a Ceast Resil Impactor, following the ISO 180 standard.

The thermal properties were analyzed using a Mettler Toledo DSC-1 Star system. The samples were tested in a nitrogen atmosphere with a heating rate of 10 °C/min over a temperature range of 25–220 °C. To eliminate the thermal history, the samples were first heated to 220 °C and held for 5 min. They were then cooled to 25 °C at a rate of 10 °C/min, followed by a second heating cycle to 220 °C at the same rate.

Thermal stability was evaluated using a Mettler Toledo TGA Q50 thermogravimetric analyzer. The tests were performed in a nitrogen atmosphere over a temperature range of 25–600 °C at a constant heating rate of 10 °C/min.

## 3. Results and Discussions

### 3.1. FTIR Results

**Figure 2:**
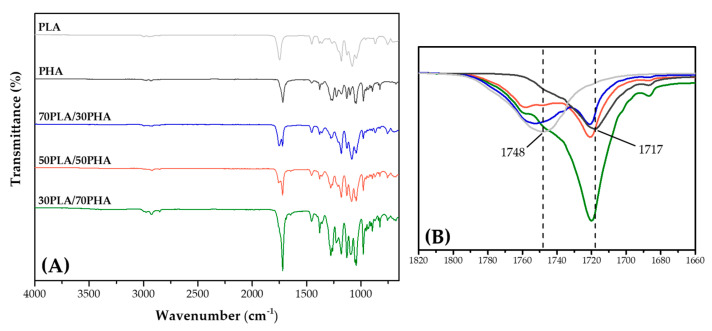
FTIR spectra of PLA, PHA, and PLA/PHA blends; (**A**) 4000–650 cm^−1^ region, (**B**) 1820–1660 cm^−1^ region.

Figure 2A presents the FTIR spectra of pure polymers (PLA and PHA), PLA/PHA blends, and their corresponding individual components over the wavenumber range of 4000 to 650 cm^−1^.

In the spectrum of pure PLA, the characteristic carbonyl (C=O) stretching vibration appeared prominently at 1748 cm^−1^. The absorption bands observed at 2995 and 2945 cm^−1^ are attributed to asymmetric and symmetric stretching vibrations of methyl (CH_3_) groups, respectively. The peaks at 1452 and 1383 cm^−1^ correspond to CH_3_ asymmetric and symmetric deformation modes. The band at 1359 cm^−1^ is assigned to C-H deformation and symmetric CH_3_ deformation associated with the crystalline regions of PLA. Additionally, the band at 1266 cm^−1^ is attributed to C–O–C stretching and amorphous C-H deformation. Other notable peaks include those at 1181, 1128, 1080, and 1042 cm^−1^, corresponding to C–O–C stretching, CH_3_ rocking, further C–O–C stretching, and C–CH_3_ stretching vibrations, respectively [29,30,31,32]. For the PHA, the absorption bands in the 3000–2850 cm^−1^ range are indicative of CH_3_ and CH₂ stretching vibrations. A strong absorption peak near 1717 cm^−1^ is attributed to C=O stretching modes. Additionally, peaks at 1275 and 1054 cm^−1^ are associated with C–O–C stretching vibrations from crystalline moieties. A prominent band near 1180 cm^−1^ corresponds to the amorphous phase C–O–C stretching vibration, characteristic of disordered polymer regions [29,33].

As illustrated in Figure 2A, the FTIR spectra of the PLA/PHA blends exhibited the characteristic absorption bands of both PLA and PHA, confirming the presence of functional groups from each polymer in the blends. However, notable spectral changes were observed in the blends, indicating molecular-level interactions between the two components. Specifically, in the carbonyl stretching region (1820–1660 cm^−1^), as shown in the magnified view in Figure 2B, shifts in the C=O stretching bands were detected. For instance, the C=O stretching band of PLA, originally located at 1748 cm^−1^ in pure PHA, shifted to 1753 cm^−1^, 1758 cm^−1^, and 1759 cm^−1^ for blend ratios of 70/30, 50/50, and 30/70 (PLA/PHA), respectively. Similarly, the C=O band associated with PHA, initially observed at 1717 cm^−1^, exhibited a shift of approximately 3 cm^−1^ in the blend spectra. These spectral shifts in the carbonyl region suggested specific interactions between the PLA and PHA molecular chains, such as transesterification, dipole–dipole interaction, etc. [29,34,35].

**Figure 3:**
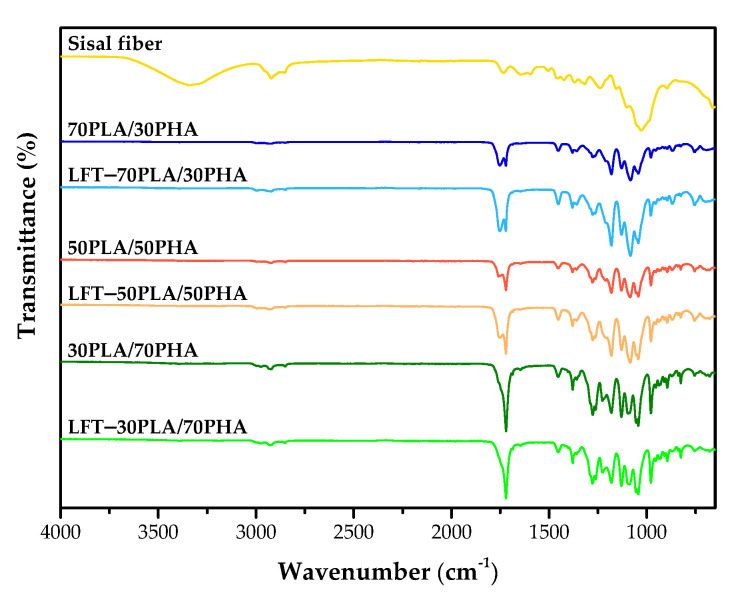
FTIR spectra of sisal fiber, PLA/PHA blends, and LFT-PLA/PHA biocomposites.

Figure 3 displays the FTIR spectra of untreated sisal fiber, PLA/PHA blends, and LFT-PLA/PHA biocomposites. The spectrum of untreated sisal fiber exhibited a broad absorption band at 3337 cm^−1^, corresponding to the O–H stretching vibrations of hydroxyl groups in cellulose. The bands observed at 2922 cm^−1^ and 2853 cm^−1^ were attributed to the C–H stretching vibrations of aliphatic groups present in cellulose, hemicellulose, and lignin. A prominent peak at 1734 cm^−1^ is associated with the carbonyl stretching of acetyl and ester groups in hemicellulose, as well as aromatic components in lignin. Additionally, the absorption band around 1646 cm^−1^ corresponds to O–H bending vibrations due to moisture absorbed within the cellulose structure. Peaks at 1594 cm^−1^ and 1503 cm^−1^ were linked to the symmetric C–C stretching vibrations of the aromatic rings in lignin [36,37,38,39]. The absorption bands at 1027 cm^−1^ and 895 cm^−1^ were indicative of C–H stretching of hydroxyl groups in cellulose and the β-glycosidic linkages between monosaccharide units in the sisal fibers [40].

Importantly, no significant shift in the characteristic absorption bands was observed upon the incorporation of sisal fibers into PLA/PHA blends, indicating that no substantial chemical interaction occurred between the fiber and polymer matrix under the conditions studied.

### 3.2. Rheological Properties

**Figure 4:**
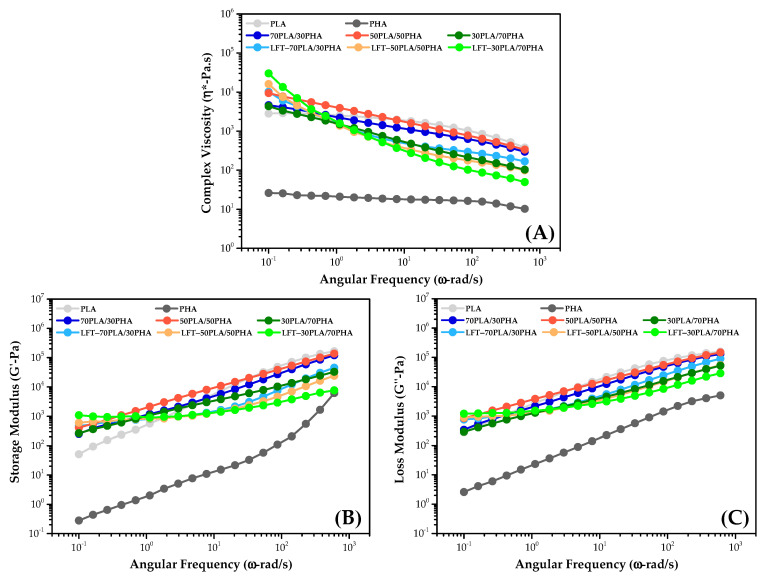
(**A**) Complex viscosity, (**B**) storage modulus, (**C**) loss modulus versus angular frequency of samples.

Figure 4 presents the complex viscosity, as well as the storage and loss modulus values, for the PLA/PHA blends and LFT-PLA/PHA composites.

Both PLA and PHA exhibited Newtonian behavior at low angular frequencies. However, as the angular frequency increases, a general decline in viscosity is observed, indicating pseudoplastic (shear-thinning) behavior [29,41]. PLA/PHA blends also display shear-thinning characteristics. Notably, the addition of PHA to PLA resulted in an increase in complex viscosity at low angular frequencies, whereas at higher frequencies, the viscosity decreases. This reduction was primarily attributed to the lower molecular weight of PHA relative to PLA [29]. Despite this, the complex viscosities of PLA/PHA blends remain higher than those of pure PLA at lower frequencies, likely due to partial interactions between the two polymers. Furthermore, even in a 50PLA/50PHA blend, the complex viscosity values were closer to those of pure PLA, suggesting that PLA acts as the continuous phase, while PHA forms the dispersed phase. In terms of viscoelastic properties, both the storage (G′) and loss (G′′) modulus values increase with rising angular frequency, which is a characteristic response of linear polymer systems [42].

It was observed that LFT-biocomposites exhibited higher complex viscosity values than PLA/PHA blends at low angular frequencies, while complex viscosity values remained below PLA/PHA blends with increasing angular frequency. At low frequencies, the presence of sisal fibers disrupts the polymer flow and restricts the mobility of polymer chain segments along the flow direction [43]. However, as the angular frequency increases, the fiber–matrix interaction weakens, and fiber alignment in the flow direction contributes to a reduction in complex viscosity. This effect becomes more pronounced with increasing PHA content in the LFT-PLA/PHA biocomposites, which is due to the lower molecular weight of PHA than PLA. Similar behavior was observed in storage and loss modulus values, which is an indication of diminished interfacial interaction between the sisal fiber and the PLA/PHA matrix.

### 3.3. Fiber Length Measurements

**Figure 5:**
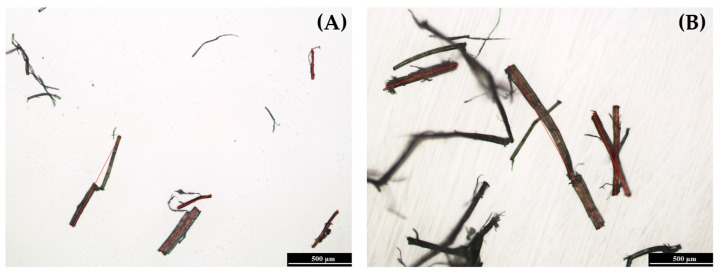
Fiber length measurement images of sisal fibers: (**A**) sdamaged fiber, (**B**) protected fiber.

**Figure 6:**
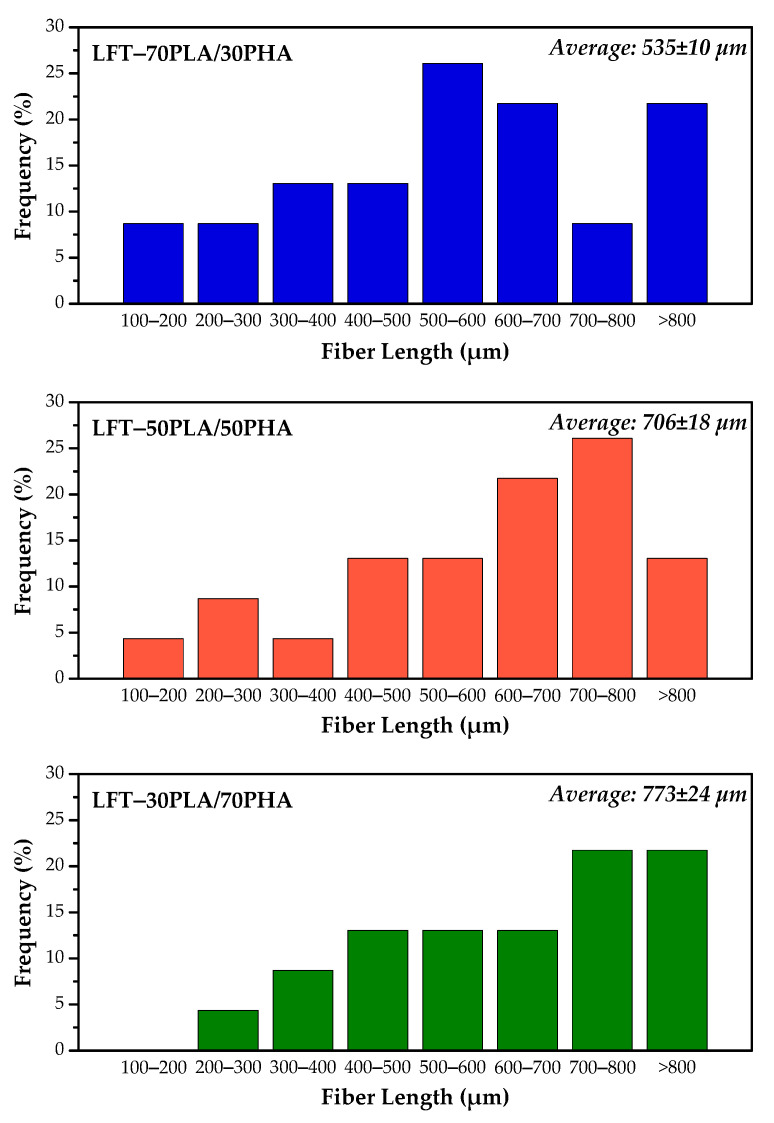
Fiber length distribution of samples.

Figure 5 illustrates representative images of fibers classified as “damaged” and “protected”. Figure 6 presents the results of the length measurements of the sisal fibers in the LFT–PLA/PHA biocomposites.

The initial length of the sisal fibers prior to compounding was approximately 1.5–2.5 cm. In the case of the LFT-70PLA/30PHA biocomposite, short and severely degraded fibers were observed, with an average length of 535 µm (Figure 5A). For the LFT-50PLA/50PHA and LFT-30PLA/70PHA biocomposites, the measured fiber lengths were 706 µm and 773 µm, respectively (Figure 6). Notably, the LFT-30PLA/70PHA formulation retained longer fiber lengths (Figure 5B), suggesting reduced fiber degradation during processing and indicating that the fibers were relatively better protected.

During processing, particularly during injection molding or extrusion, the material is subjected to high pressures, which induce shear stress. This can lead to fiber breakage and splitting, rather than facilitating fiber alignment [44,45]. The observed increase in fiber length with higher PHA content in the biocomposite can be attributed to the decrease in complex viscosity, as indicated by the rheological properties. As the amount of PHA increases, the shear forces encountered during extrusion are reduced, allowing for better fiber retention and preservation of longer fibers.

### 3.4. Scanning Electron Microscopy (SEM)

**Figure 7:**
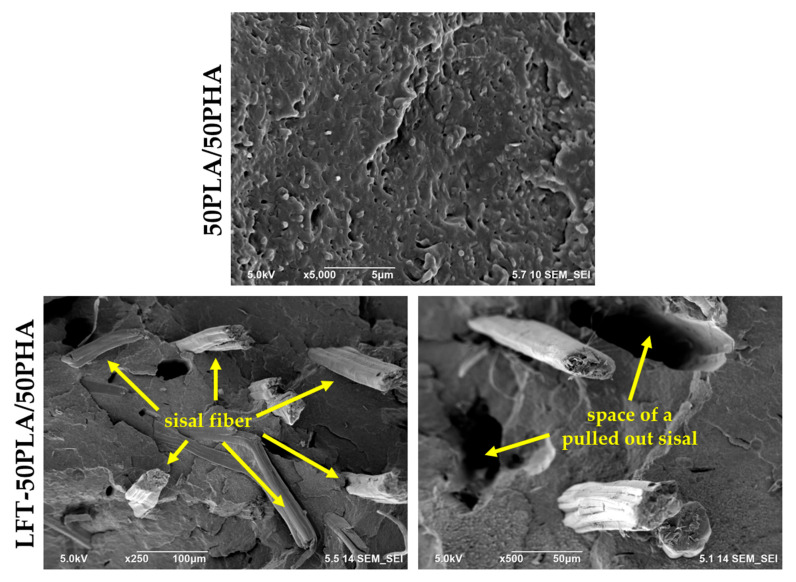
Cryogenically fractured surface morphologies of 50PLA/50PHA and LFT-50PLA/50PHA samples.

SEM analyses were conducted to investigate the dispersion of sisal fibers within the LFT-biocomposites. Examination of the SEM image for the 50PLA/50PHA blend revealed that the dispersed phase was not distinctly defined, and the average particle size of the dispersed domains is relatively small (Figure 7). This observation supported the presence of partial interaction between PLA and PHA, as previously indicated by the FTIR and rheological analysis results.

In the SEM images of the LFT-50PLA/50PHA biocomposites, it was observed that the sisal fibers were oriented along the injection direction and exhibited a homogeneous dispersion throughout the matrix. However, due to the absence of significant interfacial adhesion between the sisal fibers and the PLA/PHA polymer blend, the fibers failed to adequately bond with the matrix, resulting in the formation of interfacial gaps. Consequently, debonding of sisal fibers was observed.

### 3.5. Mechanical Properties

**Figure 8:**
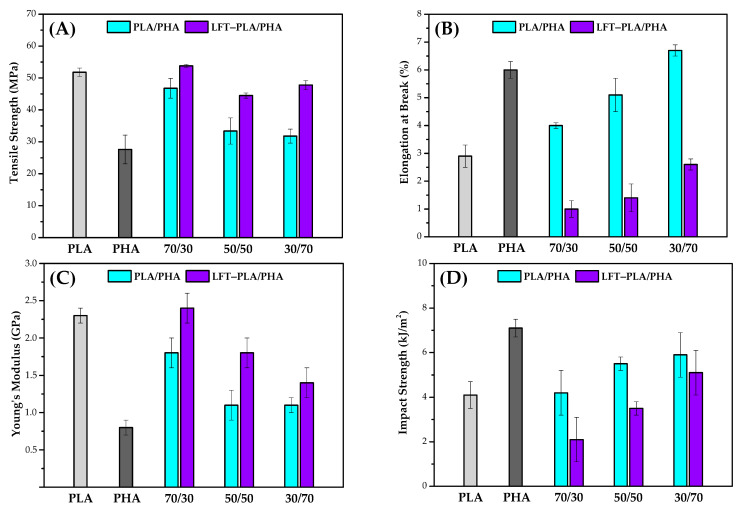
(**A**) Tensile strength, (**B**) elongation at break, (**C**) Young’s modulus, (**D**) impact strength of samples.

To assess the mechanical performance of PLA/PHA blends and LFT-PLA/PHA biocomposites, tensile and Izod impact tests were conducted. The resulting variations in mechanical properties are illustrated in Figure 8 and summarized in Table 1.

Pure PLA exhibited a tensile strength of 51.8 MPa, while the tensile strength of pure PHA was measured at 27.6 MPa. In terms of stiffness, PLA demonstrated a significantly higher Young’s modulus (2.3 GPa) compared to PHA. Upon incorporation of PHA into PLA, both tensile strength and Young’s modulus decreased, whereas the elongation at break showed a notable improvement. For instance, when 50 wt.% PHA was added to PLA, the tensile strength decreased from 51.8 MPa to 33.4 MPa. However, this reduction in strength was accompanied by an increase in elongation at break from 2.9% to 5.1%. The observed reduction in mechanical properties with increasing PHA content is attributed primarily to limited interfacial compatibility between PLA and PHA, which hinders efficient stress transfer across the polymer matrix [46,47].

The tensile strength values of LFT-PLA/PHA biocomposites were found to be higher than those of the corresponding PLA/PHA blends. Notably, the increase in tensile strength for fiber-reinforced composites with higher PLA content was approximately 15%, whereas the LFT-30PLA/70PHA biocomposite exhibited an enhancement of up to 50%. This substantial improvement in the latter is attributed to the greater preservation of sisal fiber length during extrusion, which is facilitated by the higher PHA content. The reduced shear forces associated with increased PHA content contribute to decreased fiber breakage, thereby enhancing the reinforcing efficiency of the fibers within the composite matrix. The enhancement in Young’s modulus observed in the LFT-PLA/PHA biocomposites can be attributed to the high intrinsic stiffness of the sisal fibers incorporated into the polymer matrix. The elongation at break values decreased when the PLA/PHA blends were converted into LFT-biocomposites through the incorporation of sisal fibers. This reduction in ductility is primarily attributed to the formation of stress concentration zones at the fiber ends and along the fiber–matrix interfaces. These localized stress concentrations hinder the polymer chains’ ability to undergo plastic deformation, thereby limiting the elongation behavior of the biocomposite [48,49].

The Izod impact strength of pure PLA was measured at 4.1 kJ/m^2^, while pure PHA exhibited a higher value of 7.1 kJ/m^2^. Incorporating increasing amounts of PHA into PLA resulted in a progressive enhancement of impact strength, indicating an improvement in the overall toughness of the blend. This increase is a result of the prolongation of crack pathways due to the formation of a second phase in the matrix by adding PHA.

The toughness of fiber-reinforced polymeric composites is primarily governed by interfacial characteristics and the predominant failure mechanisms occurring between the matrix and the reinforcing fibers, such as fiber breakage, pull-out, and debonding [50]. In this study, LFT-PLA/PHA biocomposites exhibited lower impact strength values compared to the corresponding PLA/PHA blends. This reduction in impact performance can be attributed to two primary mechanisms. First, the presence of fibers restricts the deformation and ductile mobility of the polymer chains, thereby limiting the material’s capacity to dissipate energy during crack propagation. Second, fibers can introduce localized stress concentrations within the composite, which lower the energy threshold required for crack initiation. These stress-concentrated zones typically occur at fiber ends, in regions with poor interfacial adhesion, or at sites where fibers are in close proximity or contact with one another [51,52].

### 3.6. DSC Results

**Figure 9:**
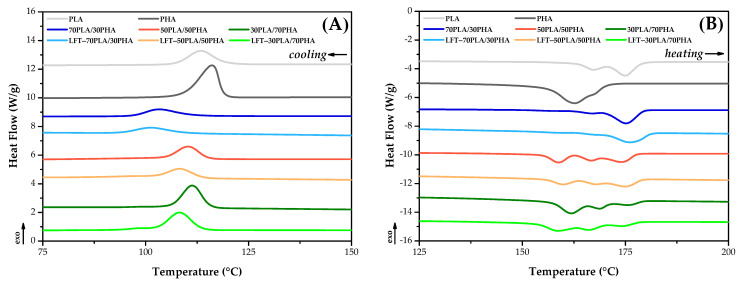
DSC thermograms of samples: (**A**) cooling, (**B**) second heating.

The thermal transitions of PLA/PHA blends and LFT-PLA/PHA biocomposites were investigated through DSC analysis. The corresponding cooling and second heating thermograms are presented in Figure 9, while the thermal transition data derived from these analyses are summarized in Table 2.

From the cooling curves, the melt crystallization peak temperatures of PLA and PHA were determined to be 113.4 °C and 116.1 °C, respectively. Upon blending PHA with PLA, a single crystallization peak was observed rather than distinct peaks for each polymer, indicating the co-crystallization of the two polymers. Furthermore, both the enthalpy of crystallization and the melt crystallization peak temperature increased proportionally with the PHA content in the PLA/PHA blends.

Analysis of the second heating thermograms revealed that PHA exhibited a single melting peak at 162.6 °C, while PLA demonstrated bimodal melting behavior with peaks at 167.2 °C and 174.9 °C. The melting enthalpies of PLA and PHA were determined to be 56.5 J/g and 104.1 J/g, respectively, suggesting that PHA possesses a more regular spherulitic structure compared to PLA. In the PLA/PHA blends, three distinct melting peaks corresponding to PLA and PHA were observed. Additionally, the melting enthalpy values of the blends increased with higher PHA content, indicating enhanced crystalline organization.

Lower melting enthalpy values were observed for the bio-LFT samples compared to the PLA/PHA blends. This reduction is attributed to the location of sisal fibers between polymer chains, which hinders chain packing and the formation of highly ordered crystalline structures. Furthermore, in the LFT-70PLA/30PHA biocomposite, a 17% decrease in melting enthalpy was recorded with sisal fiber reinforcement, whereas the LFT-30PLA/70PHA biocomposite, which has the highest PHA content, exhibited only a 4% decrease. This trend can be explained by the higher PLA content leading to shorter sisal fibers during processing, which in turn are more effective at disrupting the formation of regular spherulitic structures. In addition, the melt crystallization enthalpy of LFT-PLA/PHA was similar to that of the PLA/PHA blend. This indicated that the addition of sisal fiber did not significantly influence the crystal structure of the polymer matrix.

### 3.7. TGA Results

**Figure 10:**
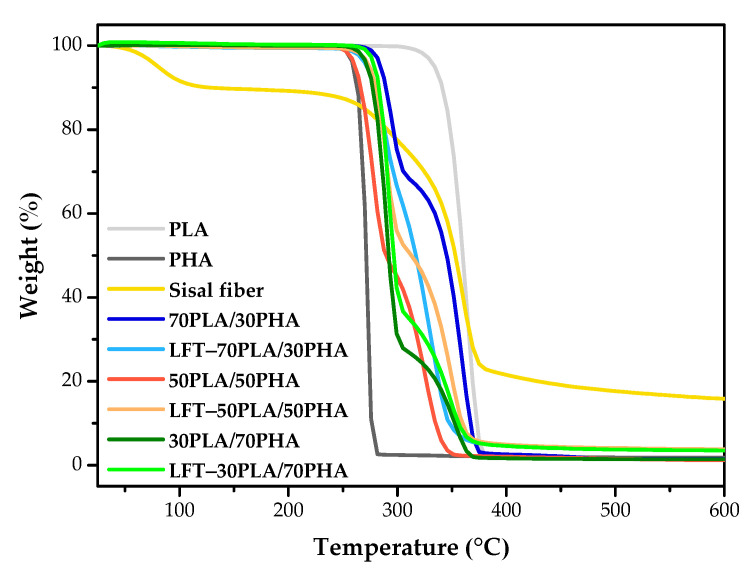
TGA curves of samples.

The thermal stability of PLA/PHA and LFT-PLA/PHA samples was assessed using thermogravimetric analysis. The TGA curves and corresponding thermal degradation data are presented in Figure 10 and Table 3, respectively. The degradation temperatures T_d5_, T_d10_, T_dmax1_, and T_dmax2_ represent the temperatures at which 5%, 10%, and maximum weight losses occurred. As illustrated in Figure 10, pure PLA and PHA samples displayed a single-step degradation behavior. The temperature corresponding to 5% weight loss (T_d5_) is 334.2 °C for PLA and 259.8 °C for PHA (Table 3), indicating that PLA exhibits superior thermal stability compared to PHA. In contrast, PLA/PHA blends displayed a two-step degradation behavior. T_dmax1_ occurred within the range of 275–295 °C, while T_dmax2_ occurred in the range of approximately 325–360 °C. The initial degradation step corresponded to the decomposition of the PHA phase, which proceeds via random chain scission mechanisms. The subsequent degradation step could be attributed to the thermal decomposition of PLA, which involves the breaking of backbone bonds and leads to the formation of cyclic oligomers, lactides, and carbon monoxide as degradation products [53,54,55]. The incorporation of PHA into PLA resulted in increased degradation temperatures at 5% and 10% weight losses, as well as an elevated T_dmax2_, suggesting enhanced thermal resistance. Additionally, the T_dmax1_ value also exhibited an upward shift.

The degradation behavior of sisal fibers showed an initial weight loss up to approximately 150 °C, which was due to the evaporation of moisture from the fiber structure. Subsequent degradation observed at 180–400 °C is primarily associated with the thermal decomposition of hemicelluloses and other low molecular weight constituents such as pectins and waxes [56,57]. The char yield of 15.8% observed in sisal fibers was attributed to the thermal condensation of lignin and the subsequent formation of stable aromatic compounds under a nitrogen atmosphere [58,59].

The degradation temperatures of LFT-PLA/PHA biocomposites increased at PHA loading levels of 50 wt.% and 70 wt.%, which can be attributed to the higher thermal stability of sisal fibers compared to pure PHA. However, the char content of the biocomposites at 600 °C also increased, reflecting the inherently high char content of the sisal fibers.

## 4. Conclusions

In this study, sisal fiber-reinforced PLA/PHA bio-LFT composites were successfully developed using a novel micro-processing technique. The incorporation of sisal fibers improved tensile strength and Young’s modulus, particularly at higher PHA contents, where fiber breakage during processing was minimized. Rheological studies confirmed the shear-thinning behavior of all the systems, with fiber incorporation leading to higher complex viscosities at low frequencies. Thermal analysis through DSC and TGA revealed a suppressed crystallization behavior and improved thermal stability with fiber reinforcement. The SEM analyses showed good fiber dispersion but indicated limited interfacial bonding, contributing to reduced elongation and impact strength. As presented in Table 4, the mechanical properties of the bio-LFT composites developed in this study are generally comparable to those of biocomposites reinforced with various natural fibers reported in the literature. Furthermore, the results indicated that increasing the PLA content in the bio-LFT composites enhances their mechanical performance. These findings suggest that the mechanical properties of bio-LFT composites have the potential for further improvement through material optimization. Interfacial adhesion between the sisal fibers and the PLA/PHA matrix and the compatibility between PLA and PHA are critical factors influencing the mechanical performance of the composites. In the current study, the primary objective was to validate the bio-LFT processing route using untreated natural fibers; therefore, no coupling agents were incorporated. To address this limitation, future work will focus on enhancing interfacial compatibility through a range of fiber surface modification techniques, including alkali treatment (mercerization), dewaxing, silane functionalization, acylation, and peroxide treatment. These strategies are anticipated to improve interfacial adhesion, thereby facilitating more efficient stress transfer and enhancing the overall mechanical performance of the composites. Overall, the bio-LFT composites demonstrated promising mechanical and thermal properties, positioning them as potential candidates for sustainable and high-performance applications, especially in the automotive and transportation industries.

## Figures and Tables

**Table 1:** Mechanical properties of samples.

Sample	Tensile Strength (MPa)	Elongation at Break (%)	Young’s Modulus (GPa)	Impact Strength (kJ/m^2^)
PLA	51.8 ± 1.3	2.9 ± 0.4	2.3 ± 0.1	4.1 ± 0.6
PHA	27.6 ± 4.5	6.0 ± 0.3	0.8 ± 0.1	7.1 ± 0.4
70PLA/30PHA	46.8 ± 3.1	4.0 ± 0.1	1.8 ± 0.2	4.2 ± 1.0
LFT-70PLA/30PHA	53.8 ± 0.5	1.0 ± 0.3	2.4 ± 0.2	2.1 ± 1.0
50PLA/50PHA	33.4 ± 4.1	5.1 ± 0.6	1.1 ± 0.2	5.5 ± 0.3
LFT-50PLA/50PHA	44.5 ± 0.8	1.4 ± 0.5	1.8 ± 0.2	3.5 ± 0.3
30PLA/70PHA	31.8 ± 2.2	6.7 ± 0.3	1.1 ± 0.1	5.9 ± 1.0
LFT-30PLA/70PHA	47.8 ± 1.4	2.6 ± 0.2	1.4 ± 0.2	5.1 ± 1.0

**Table 2:** DSC results of samples.

Sample	Cooling				Second Heating			
	T_c,onset_ (°C)	T_c,peak_ (°C)	T_c,endset_ (°C)	ΔH_c_ (J/g)	T_m1_ (°C)	T_m2_ (°C)	T_m3_ (°C)	ΔH_m_ (J/g)
PLA	125.1	113.4	102.2	49.0	---	167.2	174.9	56.5
PHA	122.1	116.1	101.6	89.5	162.6	---	---	104.1
70PLA/30PHA	115.3	103.4	93.5	26.6	156.2	167.0	176.4	45.8
LFT-70PLA/30PHA	113.1	101.3	91.5	23.0	158.4	167.1	175.2	37.8
50PLA/50PHA	118.5	110.2	101.2	40.2	158.8	166.8	174.3	57.2
LFT-50PLA/50PHA	116.8	108.4	94.1	39.0	159.8	167.9	175.2	49.0
30PLA/70PHA	119.7	111.3	96.0	58.5	162.0	168.7	176.0	66.3
LFT-30PLA/70PHA	118.4	108.3	92.2	55.4	158.5	166.2	174.6	64.0

**Table 3:** TGA results of samples.

Sample	T_d5_ (°C)	T_d10_ (°C)	T_dmax1_ (°C)	T_dmax2_ (°C)	Char Yield (%)
PLA	334.2	341.3	363.0	---	1.4
PHA	259.8	263.3	271.3	---	1.8
Sisal fiber	80.5	128.9	359.6	---	15.8
70PLA/30PHA	284.8	289.5	294.0	358.8	1.5
LFT-70PLA/30PHA	272.2	279.3	289.8	331.0	3.6
50PLA/50PHA	261.5	266.5	277.6	326.0	1.2
LFT-50PLA/50PHA	274.5	280.4	289.4	349.1	3.8
30PLA/70PHA	272.7	277.6	289.4	352.9	1.4
LFT-30PLA/70PHA	279.4	283.3	291.9	358.3	3.5

**Table 4:** Comparison of the mechanical test results from this study with those reported in the literature.

Materials	Tensile Strength (MPa)	Elongation at Break (%)	Young’s Modulus (GPa)	Impact Strength (kJ/m^2^)	Reference
LFT-70PLA/30PHA	53.8 ± 0.5	1.0 ± 0.3	2.4 ± 0.2	2.1 ± 1.0	This work
LFT-50PLA/50PHA	44.5 ± 0.8	1.4 ± 0.5	1.8 ± 0.2	3.5 ± 0.3	This work
LFT-30PLA/70PHA	47.8 ± 1.4	2.6 ± 0.2	1.4 ± 0.2	5.1 ± 1.0	This work
70PLA/30PHA/10 cellulosic fiber	44.2 ± 1.4	2.7 ± 0.3	4.3 ± 0.4	---	[60]
PLA/10 flax	60	---	2.7	17	[61]
PLA/10 jute	51	3.7	1.2	---	[62]
PLA/20 banana fiber	53.8	1.3	2.4	---	[63]
PLA/30 ramie	53	5.3	---	10	[64]
PLA/30 jute	51	5.5	---	9.2	[64]
PHB/10 hemp	16	---	---	---	[65]
PHB/10 jute	14	---	---	---	[65]

## Data Availability

The data presented in this study are available on request from the corresponding author.
